# How to achieve trustworthy artificial intelligence for health

**DOI:** 10.2471/BLT.19.237289

**Published:** 2020-01-27

**Authors:** Kristine Bærøe, Ainar Miyata-Sturm, Edmund Henden

**Affiliations:** a Department of Global Public Health and Primary Care, University of Bergen, PO Box 7804, N-5020 Bergen, Norway.; b Oslo Metropolitan University, Oslo, Norway.

## Abstract

Artificial intelligence holds great promise in terms of beneficial, accurate and effective preventive and curative interventions. At the same time, there is also awareness of potential risks and harm that may be caused by unregulated developments of artificial intelligence. Guiding principles are being developed around the world to foster trustworthy development and application of artificial intelligence systems. These guidelines can support developers and governing authorities when making decisions about the use of artificial intelligence. The High-Level Expert Group on Artificial Intelligence set up by the European Commission launched the report *Ethical guidelines for trustworthy artificial intelligence *in**2019. The report aims to contribute to reflections and the discussion on the ethics of artificial intelligence technologies also beyond the countries of the European Union (EU). In this paper, we use the global health sector as a case and argue that the EU’s guidance leaves too much room for local, contextualized discretion for it to foster trustworthy artificial intelligence globally. We point to the urgency of shared globalized efforts to safeguard against the potential harms of artificial intelligence technologies in health care.

## Résumé

L'intelligence artificielle regorge de potentiel en matière d'interventions préventives et curatives précises, efficaces et bénéfiques. Mais par la même occasion, elle présente certains risques et peut s'avérer nocive si son développement n'est pas encadré par des règles. Partout dans le monde, des principes directeurs sont instaurés afin de promouvoir un niveau de fiabilité optimal dans l'évolution et l'application des systèmes basés sur l'intelligence artificielle. Ces principes peuvent aider les développeurs et les autorités gouvernementales à prendre des décisions relatives à l'intelligence artificielle. Le Groupe d'experts de haut niveau sur l'intelligence artificielle créé par la Commission européenne a récemment publié un rapport intitulé *Lignes directrices en matière d'éthique pour une IA digne de confiance*. Objectif de ce rapport : contribuer aux réflexions et discussions portant sur l'éthique des technologies fondées sur l'intelligence artificielle, y compris dans les pays n'appartenant pas à l'Union européenne (UE). Dans ce document, nous utilisons le secteur mondial de la santé comme exemple et estimons que les directives de l'UE accordent un pouvoir discrétionnaire trop important aux autorités locales et au contexte pour véritablement encourager la fiabilité de l'intelligence artificielle dans le monde. Nous insistons également sur l'urgence de mettre en place une protection globale commune contre les éventuels préjudices liés aux technologies d'intelligence artificielle dans le domaine des soins de santé.

## Resumen

La inteligencia artificial es muy prometedora en términos de intervenciones preventivas y curativas beneficiosas, precisas y eficaces. Al mismo tiempo, también hay conciencia de los posibles riesgos y daños que pueden causar los desarrollos no regulados de la inteligencia artificial. Se están elaborando principios fundamentales en todo el mundo para fomentar el desarrollo y la aplicación confiables de los sistemas de inteligencia artificial. Estas directrices pueden servir de apoyo a los desarrolladores y a las autoridades gobernantes en la toma de decisiones sobre el uso de la inteligencia artificial. El Grupo de Expertos de Alto Nivel sobre Inteligencia Artificial establecido por la Comisión Europea ha publicado recientemente el informe *Ethical guidelines for trustworthy artificial intelligence* (Directrices éticas para una inteligencia artificial confiable). El informe tiene por objeto contribuir a la reflexión y el debate sobre la ética de las tecnologías de inteligencia artificial incluso más allá de los países de la Unión Europea (UE). En este documento, se recurre al sector sanitario mundial como caso de referencia y se argumenta que las directrices de la UE conceden demasiado margen a la discreción local y contextualizada como para fomentar una inteligencia artificial confiable a nivel mundial. Se destaca la urgencia de compartir los esfuerzos internacionales para protegerse de los posibles daños de las tecnologías de inteligencia artificial en la atención sanitaria.

## ملخص

يجمل الذكاء الاصطناعي بين طياته وعوداً جمة فيما يتعلق بالتدخلات الوقائية والعلاجية المفيدة والدقيقة والفعالة. وفي الوقت ذاته، هناك أيضًا وعي بالمخاطر والأضرار المحتملة التي قد تحدث بسبب التطورات غير المنظمة للذكاء الاصطناعي. يتم حالياً تطوير مبادئ توجيهية حول العالم لتعزيز تنمية جديرة بالثقة، وتطبيق أنظمة الذكاء الاصطناعي. يمكن لهذه الإرشادات أن تدعم المطورين والسلطات الحاكمة عند اتخاذ القرارات بشأن استخدام الذكاء الاصطناعي. قامت مؤخراً مجموعة خبراء الذكاء الاصطناعي رفيعي المستوى، التي أسستها المفوضية الأوروبية، بإصدار تقرير بعنوان *المبادئ التوجيهية الأخلاقية للذكاء الاصطناعي الجدير بالثقة *. يهدف التقرير إلى المساهمة في الأفكار والمناقشة الخاصة بأخلاقيات تقنيات الذكاء الاصطناعي كذلك خارج دول الاتحاد الأوروبي (EU). وفي هذه الورقة، نحن نستخدم قطاع الصحة العالمي كحالة، ونؤمن بأن توجيهات الاتحاد الأوروبي تترك مجالاً كبيراً لتقدير محلي مقترن بالسياق لتعزيز ذكاءً اصطناعياً جدير بالثقة على مستوى العالم. كما نشير إلى الحاجة الملحة لبذل جهود عالمية مشتركة للوقاية من الأضرار المحتملة لتكنولوجيات الذكاء الاصطناعي في الرعاية الصحية.

## 摘要

人工智能在有益、精准和有效的预防与治疗干预方面具有广阔的前景。同时，人们也意识到人工智能的无度发展可能会引起潜在的风险和危害。全世界正在制定指导原则，以促进可信赖的人工智能系统的开发和应用。这些准则可以支持开发人员和管理机构制定有关人工智能用途的决策。由欧盟委员会成立的人工智能高级专家小组最近发布了*《可信赖的人工智能伦理准则》(Ethical guidelines for trustworthy artificial intelligence)* 报告。该报告旨在促进欧盟 (EU) 以外的国家也开展人工智能技术伦理方面的反思和讨论。在本文中，我们以全球卫生部门为例并且认为欧盟的准则还有很大的完善空间，可以根据当地的具体情况自行决定，在全球范围内促进可信赖的人工智能。我们指出必须立即在全球范围内共同努力，防范人工智能技术在医疗保健领域的潜在危害。

## Резюме

Искусственный интеллект открывает большие перспективы для имеющих практическую значимость, точных и эффективных мероприятий по профилактике и лечению заболеваний. В то же самое время необходимо принимать во внимание потенциальные риски и вред, которые могут быть вызваны нерегулируемым развитием искусственного интеллекта. В настоящее время идет процесс глобальной разработки руководящих принципов, способствующих надежному и безопасному формированию и применению систем искусственного интеллекта. Данные рекомендации помогут разработчикам и руководящим органам в принятии решений относительно использования искусственного интеллекта. Созданная Европейской комиссией экспертная группа высокого уровня по искусственному интеллекту недавно выпустила отчет, озаглавленный *«Этические рекомендации для надежного и безопасного использования искусственного интеллекта»*. Цель отчета — содействие процессу анализа и обсуждения этической стороны применения технологий искусственного интеллекта за пределами Европейского союза (ЕС). В этой статье авторы рассматривают в качестве примера сектор общественного здравоохранения и доказывают, что рекомендации ЕС предоставляют слишком большую свободу действий на локальном уровне в контексте имеющихся условий, что препятствует обеспечению надежного использования искусственного интеллекта во всем мире. Авторы подчеркивают настоятельную необходимость совместных глобальных усилий по защите от потенциального вреда, который может быть нанесен сфере здравоохранения в результате использования технологий искусственного интеллекта.

## Introduction

Artificial intelligence has been defined as “the part of digital technology that denotes the use of coded computer software routines with specific instructions to perform tasks for which a human brain is normally considered necessary.”^1^ The most complex connotation of the term artificial intelligence, that of machines with human-like general intelligence, is still a distant vision. However, artificial intelligence in the more restricted sense defined above is already broadly embedded in society in a variety of forms. The pace of development of new and improved artificial intelligence-based technologies is rapid; the question is no longer whether artificial intelligence will have an impact, but “by whom, how, where, and when this positive or negative impact will be felt.”^2^

Many areas of health care could benefit from the use of artificial intelligence technology. According to a recent literature review, artificial intelligence is already being used: “(1) in the assessment of risk of disease onset and in estimating treatment success [before] initiation; (2) in an attempt to manage or alleviate complications; (3) to assist with patient care during the active treatment or procedure phase; and (4) in research aimed at elucidating the pathology or mechanism of and/or the ideal treatment for a disease.”^3^ On the risk side, others have summarized several health-related concerns: the potential for bias in the data used to train artificial intelligence algorithms; the need for protection for patients’ privacy; potential mistrust of digital tools by clinicians and the general public; and ensuring health-care personnel handle artificial intelligence in a trustworthy manner.^4^ Other concerns relate to physical applications of artificial intelligence. For example, while robots could be useful in the care of the elderly, there are risks of reduced contact between humans, the deception of encouraging companionship with a machine and loss of control over a person’s own life.^5^ Questions have also been raised about the extent to which artificial intelligence technologies could replace clinicians^6^ and, if so, whether the opacity of machine learning-based decisions weaken the authority of clinicians, threaten patients’ autonomy^7^ or jeopardize shared decision-making between doctor and patient.^8^

Discussions of the risks posed by artificial intelligence systems range from current concerns, such as violations of privacy or harmful effects on society, to debates about whether machines could ever escape from human control. However, fully predicting the consequences of these technological developments is not possible. The need for a precautionary approach to artificial intelligence highlights the importance of thoughtful governance. By applying our human intelligence, we have the opportunity, through control of decision-making, to steer the development of artificial intelligence in ways that accord with human values and needs.

Guidance has been developed through initiatives that aim to foster responsible and trustworthy artificial intelligence and to mitigate unwanted consequences. Examples include AI4People,^2^ Asilomar AI principles^9^ and the Montreal Declaration for a Responsible Development of Artificial Intelligence.^10^ In this paper we focus on the report of the independent High-Level Expert Group on Artificial Intelligence set up by the European Commission.^11^ *Ethical guidelines for trustworthy AI* identified trustworthy artificial intelligence as consisting of three components: (i) compliance with all applicable laws and regulations; (ii) adherence to ethical principles and values; and (iii) promotion of technical and societal robustness. These components are important throughout the cycle of development, deployment and use of artificial intelligence. 

**Fig. 1:**
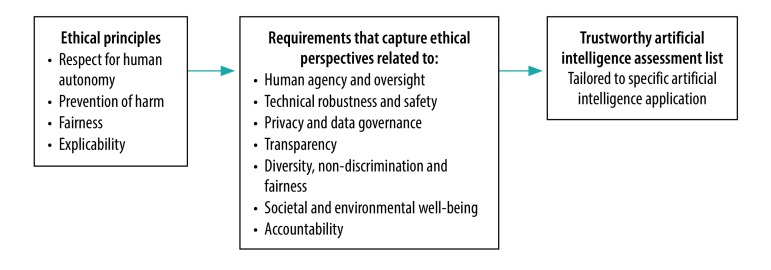
Framework for trustworthy artificial intelligence

Source: Adapted from the report of the European Commission High-Level Expert Group on Artificial Intelligence.^9^

The expert group’s report focuses on the ethics and robustness of artificial intelligence rather than the legal issues, basing ethical guidance for trustworthy artificial intelligence on a fundamental rights approach.^11^ Four principles rooted in these fundamental rights shape the framework and are translated into more specific requirements: (i) respect for human autonomy; (ii) prevention of harm; (iii) fairness; and (iv) explicability (the report stresses that this list is not necessarily exhaustive). These requirements can translate into a tailored list to allow for assessments of specific artificial intelligence interventions (Fig. 1). The first three principles are well-established in the bioethical literature.^12^ The principle of explicability is intended to gain an understanding of how artificial intelligence generates output, which is important for contesting decisions based on artificial intelligence and tracing appropriate chains of accountability.^2^

These general ethical principles may, however, conflict with each other. Any conflicts should be managed by deliberations that are accountable to and conditioned by democratic systems of public engagement and processes of open political participation.^11^ The expert group’s report strongly emphasized the need for responsible governance and the role of stakeholders “including the general public.” The safety mechanisms proposed include a “basic artificial intelligence literacy [that] should be fostered across society.” Building trustworthiness into artificial intelligence therefore relies on assumptions about a high-functioning, deliberative democracy and its governing potential to drive the development of artificial intelligence-based technologies. The expert group also highlighted the context-specific nature of artificial intelligence (“different situations raise different challenges”) and the need for an additional sectoral approach to the general framework they propose. As part of a coordinated artificial intelligence strategy for the European Commission and European Union (EU) Member States, the report’s recommendations are expected to be central in shaping the development and use of artificial intelligence in Europe. 

Since the use and impact of artificial intelligence spans national borders, the expert group calls for work towards a global ethical guidance.^11^ We believe that the ethical concerns and challenges addressed by the EU’s framework have global relevance. Basing the principles on a fundamental rights approach means that their relevance and significance can be considered universal. Indeed, the principles are rooted in the same rights and obligations that structure most of the United Nations’ (UN) sustainable development goals (SDGs) and that influence development strategies in low-and middle-income countries beyond the EU. Thus, the content of the ethical framework (principles and guidelines) fronted by the expert group can be expected to carry legitimacy across cultural contexts and economic divides. The risks and potential negative impact artificial intelligence systems can have, for example “on democracy, the rule of law and distributive justice, or on the human mind”^11^ are also applicable wherever such institutions are in place.

The EU expert group’s framework is a process of first identifying fundamental ethical principles that are acknowledged through public debate as relevant for different contexts and across domains of analysis and then translating these principles into “viable guidelines to shape artificial intelligence-based innovation.”^13^ However, while an ethical framework can recommend a process for how to resolve conflicting ethical principles in real-world situations, it cannot provide concrete, practical solutions for specific contexts. Moreover, ethical frameworks may not provide guidance on how to deal with conflicts that can occur between realizing the aim of the framework itself (such as ethically justified artificial intelligence) and realization of other goods (such as economic growth or achieving the SDGs). In the following section, we discuss how such conflicts occur in the health-care arena in ways that undermine the trustworthiness assumed by the expert group’s framework. Finally, we outline how a global initiative emerging from within various sectors can constructively promote globally trustworthy applications of artificial intelligence.

## Ethical challenges

Despite its global relevance, the EU expert group’s framework may fail to provide trustworthy safeguards for the use of artificial intelligence in all health settings. We reflect here on how general, structural features of global health systems, general human motivation and known drivers of interest for actions might together impact on the development and implementation of artificial intelligence systems. We have identified five areas of ethical concern: (i) conflicting goals; (ii) unequal contexts; (iii) risk and uncertainty; (iv) opportunity costs; and (v) democratic deficits (Box 1). These distinct concerns, when combined, demonstrate the need to foster trustworthy development, deployment and use of artificial intelligence as an explicitly global and transnational endeavour.

Box 1 **Ethical challenges for the global development and implementation of artificial intelligence systems in health care**

**Conflicting goals**

Forces, such as the economic interests of the artificial intelligence industry and the political objective of the United Nations’ sustainable development goals, can work against the promotion of ethically safe artificial intelligence technologies.

**Unequal contexts**

Unequal contextual factors across countries create different bases for the ethical assessment of acceptable employment of artificial intelligence and may thus sustain a non-universal standard and inequitable quality of health care across borders.

**Risk and uncertainty**

In lower-income countries with challenging living conditions, promises of effective artificial intelligence solutions that can improve the situation could override precautionary concerns about the potential risks.

**Opportunity costs**

The opportunity costs of replacing human intelligence with artificial intelligence has implications for the experiences that citizens bring into the political debate and the organization of powers and political institutions.

**Democratic deficits**

Many countries do not have sufficiently high-functioning, deliberative democracies in place and lack the ability to adequately manage and control the precautionary risks and societal impact assessments of artificial intelligence.

### Conflicting goals

The health sector is affected by strong forces of global political governance, as exemplified by SDG 3 to: “ensure healthy lives and promote well-being for all ages,” and target 3.8 to: “achieve universal health coverage, including financial risk protection, access to quality essential health-care services and access to safe, effective, quality and affordable essential medicines and vaccines for all.”^14^ These global forces that shape local priority-setting in health care may, however, represent conflicting goals. For example, the goals of equality of access to care and equality of care quality are not inherently connected and can conflict with each other when implemented.^15^ Another example is the efficient use of resources when deciding what to include in universal health coverage. Governance of health care such that it meets all political and cost–effectiveness aims inevitably leads to trade-offs and priority-setting. Such trade-offs become more difficult when resources are scarcer.

Application of artificial intelligence can be more cost–effective than human labour. The call for cost–effective, un-biased, equality-promoting solutions in the health sector can therefore be seen as an open invitation for constructive cooperation with the artificial intelligence industry. Tools already being discussed include personal care robots,^5^ ambient assisted living technologies^16^ and humanoid nursing robots.^17^ A potential challenge, however, is that such machines might not be able to provide the same overall quality of care for everyone, as do interactions with human beings. Increasing accessibility by implementing these more cost–effective methods will not solve the issue of equality if the quality of artificial intelligence-based interactions is worse than human interactions.

Another consequence of striving to reach political goals needs to be addressed. The global challenge of achieving universal access to health care provides the artificial intelligence industry with an opportunity not only to promote a societal good, but also to make favourable investments. Yet if the profits are not fairly distributed, these investments will only benefit the artificial intelligence industry economically and thereby contribute to accumulated wealth for some. The inequalities in living conditions that are associated with health inequalities may therefore persist or even worsen, thus undermining the political goal of ensuring healthy lives. Redistributing the economic benefits of large-scale investments in artificial intelligence in public health could be one way of compensating for the adverse determinants of health and therefore a strategy for promoting overall health equality. Transnational regulations are, however, required for such a redistribution to occur in a systematic manner within and across higher- and lower-income countries.

Finally, the demand for policy decisions in the health sector to be based on empirical evidence is also a force that may influence decisions to employ artificial intelligence technologies in the health-care sector and may distract from the risks associated with artificial intelligence technologies. The empirical evidence on which to base future risk calculations and assessments of new technologies may not be available before the opportunity to implement safeguards (such as regulations) has passed. Even when there are attempts to consider the uncertain, long-term impacts of artificial intelligence, the uncertain conclusions may be traded-off for clearly effective, short-term solutions to important problems.

### Unequal contexts

The conditions in which people live, and therefore the determinants of their health, vary across countries. Many countries will struggle to find the resources to address the SDGs and they will have to achieve the greatest possible health benefit out of the funds they have. In lower-income countries life expectancy is increasing due to decreases in infectious diseases.^18^ The prevalence of noncommunicable diseases associated with older age will likely increase. Cost–effective artificial intelligence technologies that can help reach, screen, diagnose, prescribe treatment for and even care for such patients will be invaluable where there are insufficient resources to increase the health workforce to meet the demographic challenge. On the other hand, in a high-income country with a well-developed, publicly funded health-care system introducing artificial intelligence-based methods to, for example, follow up the day-to-day social and nutritional care of elderly people could create other concerns. For example, the artificial intelligence system might replace an established, well-functioning workforce, which raises the concern that something valuable is lost in that transition. Unequal contextual factors create a different basis for the ethical assessment of the appropriateness of artificial intelligence. If applications of artificial intelligence actually provide poorer quality care for the elderly than does human intelligence, then there is a risk of accepting different ethical standards for higher- and lower-income countries within the same ethical framework and thereby sustaining inequitable quality of health care across borders.

### Risk and uncertainty

SDG 3 establishes an urgency to the goals of promoting health and reducing health inequalities, while measuring countries on how they perform contributes another layer of motivation. Yet being willing to risk more to achieve aims to which strong values are attached creates a structural dilemma in the area of health and artificial intelligence ethics. When resources are scarce, there might be a willingness to discount potential future harms. For example, implementing resource-efficient digital tools to monitor the movements of people with dementia could be seen as a step towards greater surveillance of society in the future. When people are in need of health care, concerns about the uncertain, potentially problematic, long-term impact of receiving help from an artificial intelligence-based system might not be their main priority. On a political level, precautionary thinking about highly uncertain future impacts may be ignored in favour of an effective solution, which helps to solve a national health challenge. Furthermore, tolerance towards the potential unwanted consequences of implementing artificial intelligence will likely depend on how intolerable the current state of affairs is perceived to be.

### Opportunity costs

In the health-care sector, we need to consider the opportunity cost of not implementing potentially beneficial artificial intelligence technology.^2^ There are, however, specific opportunity costs of choosing artificial intelligence at the expense of human intelligence. The implementation of artificial intelligence in health care might gradually replace or complement functions previously performed by human intelligence, such as exercising clinical judgement and providing assistance for those who need the help of others. The consequences for the workforce due to such replacement must be considered on the path to the SDGs. The input of human judgement will move further up the chain of decision-making, even when the EU’s ethical guidance is adhered to and artificial intelligence is overseen and controlled by human beings. The developers and managers of artificial intelligence will therefore become the authorities on the value and trade-offs involved in artificial intelligence decisions. This transition into a more centralized control of health care might create less autonomy for the remaining health-care professionals and thus negatively impact their own health. Moreover, if human-to-human interactions are replaced by human-to-artificial intelligence interactions, opportunities for the valuable, health-promoting benefits of human interactions, such as emotional intimacy, reassurance and affirmation of self-worth through others, will decrease.

Another concern is that a decrease in the health-care workforce means having fewer people (as stakeholders) who can feed their experiences with fellow humans’ social and health-care needs into processes of public, political deliberation. Open democratic discussions are important safeguards against the undesirable outcomes of artificial intelligence technology. A feedback loop is therefore created wherein increasing implementation of artificial intelligence methods leads to even weaker safeguards. Loss of the educated workforce in health (and other sectors) means that the key elements of the deliberative process to establish safety mechanisms for artificial intelligence technologies can therefore be lost.

All the above are potential opportunity costs of implementing artificial intelligence at the expense of a workforce that is driven by individual human judgement. Such lost opportunities must be identified and discussed as part of a global, self-reflective, trustworthy artificial intelligence strategy. This is in addition to the risks discussed in the EU’s expert group report.

### Democratic deficits

The EU expert group’s guidance on trustworthy artificial intelligence is based on the assumptions that high-functioning democracies and societies are present to administer and counteract or control any undesirable outcomes of artificial intelligence systems. A potential challenge, however, is that these safeguarding mechanisms and governing powers may not be in place in all countries. Also, how best to organize stakeholder involvement in the health sector is still being debated^19^^,^^20^ and current deliberations over artificial intelligence could be a new path for developing such governing institutions. Developing safety mechanisms based on particular cultural and social traditions for organizing and managing political issues would, however, be a time-consuming process. The development of artificial intelligence and the forces that drive it cannot be slowed to the pace of these deliberations.

A related, structural danger (which is not unique to artificial intelligence) is that the impact of artificial intelligence technology developed without safety restrictions in one country might affect other countries. An artificial intelligence health tool that would not even be considered in the design laboratory of one society could, however, be placed on the agenda of policy-making debates simply because it exists as an available option elsewhere.

## A global approach

The artificial intelligence industry is driven by strong economic and political interests. Gaining trustworthy control over the potential risks and harms related to artificial intelligence is therefore crucial. The EU expert group’s framework is designed to translate general principles into more concrete guidance and recommendations for how to address artificial intelligence. However, the framework does not address threats to the attainment of trustworthy artificial intelligence embedded in real-world interests and complex circumstances. Securing the governance of trustworthy artificial intelligence technologies, locally and globally, in health and other sectors, will have to be based on an expanded understanding of what translation of ethical norms into practice requires by addressing and managing structural concerns as those we have identified.

More concretely, there is a need for transnational development of shared, explicitly articulated rules that are context-independent, rather than for a framework that is too context-specific, at least before there has been a chance to develop local, protected political institutions. Low-income nations might be deterred from implementing cost–effective, but potentially unsafe artificial intelligence technologies to solve their short-term problems. As part of a global endeavour, high-income nations bear a responsibility to compensate for the potential losses to these countries, for example by financially supporting education of a scaled-up workforce of health-care personnel. The sector-specific challenges, as pointed out by the EU’s report and highlighted by our analysis, mean that targeted translation of shared general principles into specific, global regulations could guard against the potential dangers of artificial intelligence-based technology. The World Health Organization, together with the other UN bodies, is well-placed in the field of health, to lead such shared efforts towards globally trustworthy artificial intelligence.
